# Estimated Prevalence of *Cryptococcus* Antigenemia (CrAg) among HIV-Infected Adults with Advanced Immunosuppression in Namibia Justifies Routine Screening and Preemptive Treatment

**DOI:** 10.1371/journal.pone.0161830

**Published:** 2016-10-19

**Authors:** Souleymane Sawadogo, Boniface Makumbi, Anne Purfield, Christophine Ndjavera, Gram Mutandi, Andrew Maher, Francina Kaindjee-Tjituka, Jonathan E. Kaplan, Benjamin J. Park, David W. Lowrance

**Affiliations:** 1 Division of Global HIV/AIDS (DGHA), Centers for Disease Control and Prevention (CDC), Windhoek, Namibia; 2 Namibia Institute of Pathology (NIP), Windhoek, Namibia; 3 Mycotic Disease Branch, Centers for Disease Control and Prevention (CDC), Atlanta, GA, United States of America; 4 Directorate of Special Programs, Ministry of Health and Social Services, Windhoek, Namibia; 5 Division of Global HIV/AIDS (DGHA), Centers for Disease Control and Prevention (CDC), Atlanta, GA, United States of America; 6 Global Health Sciences, University of California San Francisco, San Francisco, CA, United States of America; University of New South Wales, AUSTRALIA

## Abstract

**Background:**

Cryptococcal meningitis is common and associated with high mortality among HIV infected persons. The World Health Organization recommends that routine Cryptococcal antigen (CrAg) screening in ART-naïve adults with a CD4^+^ count <100 cells/μL followed by pre-emptive antifungal therapy for CrAg-positive patients be considered where CrAg prevalence is ≥3%. The prevalence of CrAg among HIV adults in Namibia is unknown. We estimated CrAg prevalence among HIV-infected adults receiving care in Namibia for the purpose of informing routine screening strategies.

**Methods:**

The study design was cross-sectional. De-identified plasma specimens collected for routine CD4^+^ testing from HIV-infected adults enrolled in HIV care at 181 public health facilities from November 2013 to January 2014 were identified at the national reference laboratory. Remnant plasma from specimens with CD4^+^ counts <200 cells/μL were sampled and tested for CrAg using the IMMY^®^ Lateral Flow Assay. CrAg prevalence was estimated and assessed for associations with age, sex, and CD4^+^ count.

**Results:**

A total of 825 specimens were tested for CrAg. The median (IQR) age of patients from whom specimens were collected was 38 (32–46) years, 45.9% were female and 62.9% of the specimens had CD4 <100 cells/μL. CrAg prevalence was 3.3% overall and 3.9% and 2.3% among samples with CD4^+^ counts of CD4^+^<100 cells/μL and 100–200 cells/μL, respectively. CrAg positivity was significantly higher among patients with CD4+ cells/μL < 50 (7.2%, P = 0.001) relative to those with CD4 cells/μL 50–200 (2.2%).

**Conclusion:**

This is the first study to estimate CrAg prevalence among HIV-infected patients in Namibia. CrAg prevalence of ≥3.0% among patients with CD4^+^<100 cells/μL justifies routine CrAg screening and preemptive treatment among HIV-infected in Namibia in line with WHO recommendations. Patients with CD4^+^<100 cells/μL have a significantly greater risk for CrAg positivity. Revised guidelines for ART in Namibia now recommend routine screening for CrAg.

## Introduction

Cryptococcal meningitis (CM) is one of the leading opportunistic infections (OI) that is associated with high mortality among HIV-infected adults. Over 700,000 new cases of CM and 500,000 deaths from CM are estimated to occur each year in sub-Saharan Africa [1, 2]. Mortality rates of CM approach 70% in sub-Saharan Africa [3], where limited resources can lead to diagnostic delays and sub-optimal therapy [4, 5].

Cryptococcal antigenemia (CrAg) is an independent predictor of CM and death in HIV-infected individuals with severe immunosuppression [6]. CrAg is detectable in peripheral blood on average 22 days prior to development of CM and approximately 11% of people will have CrAg present > 100 days prior to disease onset [7]. Therefore, early detection of CrAg followed by preemptive treatment with fluconazole could prevent deaths from CM [8]. Targeted screening programs are likely to be most efficient among patients with low CD4^+^ cell count, which is an independent predictor of CrAg positivity [9, 10, 11, 12], In comparison, screening for CM using the current standard methods—including microscopy or fungal culture of cerebrospinal fluid obtained through lumbar puncture or detection of CrAg in body fluids via latex agglutination or enzyme immunoassay may be suboptimal. The presenting symptoms of CM (headache and fever) are usually non-specific and laboratory tests are deferred until CM disease is advanced, at which time the prognosis is poor. Therefore, the LFA presents a potentially cost-effective method for early, sub-clinical detection of CrAg and preemptive treatment to prevent overt CM among HIV-infected adults.

The World Health Organization (WHO) recommends routine CrAg screening of HIV-infected adults not yet on ART with a CD4^+^ count <100 cells/μL followed by preemptive antifungal therapy if CrAg positive, as a strategy to reduce the development of CM [13]. However, this screening strategy is recommended only if the prevalence of CrAg is ≥ 3%. Published research estimates that CrAg prevalence in sub-Saharan Africa ranges from 4% to 15% among people living with HIV (PLWHIV) [14, 15, 16].

Namibia, a sparsely populated country of approximately 2.2 million people, ranks among the highest-HIV prevalence countries in the world, with 14% of the adult population (229,631 people) living with HIV [17]. The Government of the Republic of Namibia introduced an ART program in 2002 and has achieved relatively high coverage, with approximately 80% of those in need of ART receiving it by 2014. However, HIV/AIDS is still the leading cause of mortality among adults, responsible for 2,545 deaths annually [18]. The relative burden of CM and prevalence of CrAg among HIV infected adults in Namibia is unknown. The objective of this study was to determine the prevalence of CrAg among HIV-infected adults in Namibia with CD4<200 cells/μL using the lateral flow assay (LFA) and to assess differences in CrAg prevalence among patients with CD4^+^ counts of <100 cells/μL and 100–200 cells/μL. Results from this study are intended to guide the development of routine CrAg screening strategies among HIV-infected adults in Namibia.

## Materials and Methods

A cross sectional study design was used. From November 2013 to January 2014 whole blood specimens were collected from HIV-infected adults (age ≥ 15 years) receiving HIV care at all Ministry of Health and Social Services (MOHSS) facilities countrywide for the purpose of routine CD4^+^ testing and sent to district-level Namibia Institute of Pathology (NIP) laboratories. At the time of this study, guidelines recommended CD4+ testing for all patients at staging in HIV care, at subsequent six-month intervals for those not immediately eligible for ART and at six-month intervals for monitoring patients receiving ART. Specimens collected from patients receiving ART and from ART-naïve patients were included. Following centrifugation, CD4^+^ cell counts were enumerated on whole blood specimens using (EPICS Coulter FC 500) at the NIP laboratories. Specimens with CD4^+^ counts < 200 cells/μL were identified, plasma separated and kept at 4°C until CrAg testing. Routine CD4^+^ test results were returned to ordering facilities, disclosed to patients and used to guide clinical care as per routine MOHSS procedures. Results of CrAg testing, which was not standard of care at the time of this evaluation, were not returned to facilities from which CD4 + tests were ordered and clinical outcomes of these patients were not assessed as part of this study.

### Laboratory Procedures

A LFA for rapid, “point-of-care” (POC) detection of CrAg was developed by IMMY (Immuno-Mycologics, Norman, OK, US) was used in the assessment. The LFA is a dipstick immuno-chromatographic assay. The LFA has high diagnostic accuracy [19, 20, 21], is stable at room temperature, has rapid turn-around-time and requires minimal technical skill, laboratory infrastructure and sample volume (40μl). Plasma samples were tested in batches for CrAg using the LFA. Laboratory staff were trained to perform and interpret the LFA before CrAg testing commenced. Testing was performed according to the manufacturer instructions. Briefly, one drop of the LFA specimen diluent was placed in a test tube and 40 μL of plasma was added. The CrAg test strip was then inserted into the tube and the test result was read after ten minutes. The results were interpreted according to the manufacturer instructions and recorded on a standard lab test results form.

### Data Management and Analysis

Blood specimens were delinked and de-identified prior to study-related testing so that no personal identifying information was accessible. Individual test results were immediately recorded on a laboratory data collection sheet at the time the test results became available and entered in a study-specific database after verification. Results were double-entered into the laboratory master file in turns by the same laboratory technicians who performed the tests to prevent any data entry errors. Data quality checks were performed each day by the laboratory supervisor. Demographic data were abstracted from laboratory requisition forms and linked to test results data.

Data were analyzed using Stata V.12 SE (S1 Text). Demographic and laboratory results data were described as counts, percentages and 95% confidence intervals (CI) for categorical variables and median and interquartile ranges (IQR) for continuous variables. CrAg prevalence was stratified by sex, age, and CD4^+^ test result. Standard and relative standard errors were calculated for stratified prevalence estimates. Associations between these variables and CrAg positivity were tested using the Pearson’s Chi-square test. *P*-values ≤ 0.05 were considered statistically significant.

### Ethical Review

Prior to implementation, the protocol for this study was approved by the Research Committees of the Namibia Ministry of Health and Social Services and the Namibia Institute of Pathology and the Associate Directors for Science of the National Center for Emerging and Zoonotic Infectious Diseases (NCEZID) and the Division of Global HIV AIDS (DGHA) of the US Centers for Disease Control and Prevention. Patient consent for the collection, storage, and additional testing of specimens was not required because this study did not involve contact with human subjects and blood specimens used were remnant samples collected as part of routine bio-clinical monitoring of PLWHIV and de-identified before CrAg testing.

## Results

During the sampling period, 54,530 CD4 were performed of which a total of 825 specimens (1.5%) met the study inclusion criteria and tested for CrAg. Specimens collected from females (n = 374) accounted for 45.9% of the total sample (Table 1). Data on sex, age and CD4 results were missing from 11, 12 and 12 patients respectively.

**Table 1:** Select demographic and clinical characteristics of sampled patients, sero-survey of *Cryptococcus* antigenemia among HIV-infected adults with advanced immunosuppression in Namibia, 2013–14.

Variable	Result
**Patient samples included**, *#*	825
**Sex**, *#*, *[% (95% CI)]* ^a^	
Female	374, [45.9 (42.5–49.4)]
Male	440, [54.1 (50.6–57.5)]
**Age**, *median (min—max) (IQR) years* ^b^	38 (15–94) (32–46)
**Age group**, *#*, *[% (95% CI)]*	
15–24 years	50 [6.2 (4.7–8.0)]
25–34 years	224 [27.6 (24.6–30.7)]
≥ 35 years	539 [66.3 (63.0–69.5)]
**CD4**^**+**^ **result**, *median (IQR) cells/μL* ^c^	85 (51–114)
**CD4**^**+**^ **result strata 1**, *#*, *[% (95% CI)]*	
< 100 cells/μL	511, [62.9 (59.7–66.4)]
100–200 cells/μL	302, [37.1 (33.9–40.5)]
**CD4**^**+**^ **result strata 2**, *#*, *[% (95% CI)]*	
< 50 cells/μL	194, [23.5 (20.7–26.6)]
50–200 cells/μL	630, [76.5 (73.4–79.3)]

^a.^ data missing, n = 11

^b.^ data missing, n = 12

^c.^ data missing, n = 12

The median (IQR) age of patients from whom specimens were collected was 38 (32–46) years. The majority of specimens were collected from patients’ age ≥ 35 years (66.3%). CD4^+^ results ranged from 1–198 cells/μL and the median (IQR) result was 85 (51–114) cells/μL. Specimens with a CD4^+^ result <100 cells/μL comprised 62.9% of the sample, and those with <50 cells/μL comprised 23.5%.

The prevalence of CrAg was 3.3% overall, 2.9% among females and 3.6% among males (Table 2). CrAg prevalence was 4.0% and 3.0% among patients in the 15–34 and > 35 year age groups, respectively. CrAg prevalence was 3.9% and 2.3% in the CD4 <100 cells/μL and CD4 100–200 cells/μL strata, and respectively. CrAg positivity was significantly higher among patients with CD4+ cells/μL < 50 (7.2%, *P* = 0.001) relative to those with CD4 cells/μL 50–200 (2.2%)

**Table 2:** Prevalence and correlates of CrAg positivity among HIV-infected adults with advanced immunosuppression in Namibia, 2013–14.

Variable	CrAg Prevalence (95% CI) ^a^	Standard error	Relative standard error	*P*-value
**Overall**	3.3 (2.3–4.9)	0.6	18.6	
**Sex**				
Female	2.9 (1.6–5.2)	0.9	29.7	0.58
Male	3.6 (2.2–5.9)	0.9	24.6	
**Age**				
15–34 years	4.0 (2.2–7.1)	1.2	29.6	0.43
≥ 35 years	3.0 (1.8–4.8)	0.7	24.7	
**CD4**^**+**^ **count strata 1**,				
< 100 cells/μL	3.9 (2.5–6.0)	0.9	21.9	0.22
100–200 cells/μL	2.3 (1.1–4.8)	0.9	37.4	
**CD4**^**+**^ **count strata 2**,				
< 50 cells/μL	7.2 (3.6–11.3)	1.8	24.4	0.001
50–200 cells/μL	2.2 (1.5–2.9)	0.3	14.2	

^a^. 95% confidence intervals are binomial exact, two-sided CI interval where no cases of CrAg were detected.

## Discussion

This is the first study to estimate the prevalence of CrAg among HIV-infected adults in Namibia, which is among the countries with the highest burden of HIV disease in the world. According to these findings, routine CrAg screening and preemptive treatment among HIV infected adults with CD4<100 cells/μL should be considered in Namibia, consistent with WHO normative guidance. Given the high mortality associated with HIV-related CM, our results demonstrate that routine screening for CrAg and preemptive treatment has the potential to save lives in Namibia.

According to routine ART program data from Namibia in 2014, 6,175 (36.3%) of the 17,026 patients that initiated ART had a CD4 < 200 cells/uL. Additionally, approximately 15% of patients on ART in Namibia have CD4 < 200 cells/uL at 12 months after treatment initiation. *Therefore*, *if* we apply the overall CrAg prevalence rate of 3.3% from our study, we estimate that approximately 203 patients newly initiating treatment and 75 patients already on ART for 12 months would have tested CrAg positive and been put on preemptive antifungal therapy. Without this screening program, more than 75% of these patients would likely have developed CM and been at elevated risk of death [22, 23, 24, 25].

WHO recommendations address CrAg screening in ART-naïve adults. A potential limitation of our study is that both ART-naive and ART-experienced patients were included. We were unable to distinguish between these groups of patients because data were not available on the standard laboratory requisition forms attached to the specimens we tested for CrAg. Nevertheless, inclusion of ART-experienced patients may be justified by the fact that, although the majority of patients who present with clinical CM are ART-naïve, significant CM-associated mortality also occurs within the first three months after initiating ART [26]. Furthermore, although ART modifies levels of immunosuppression, it is immunosuppression—not absence of ART—that is the factor shown in the published literature to be independently associated with CrAg positivity and CM [24, 25]. Therefore, we feel that the inclusion of some patients on ART does not diminish the practical implications of our findings of high overall CrAg positivity among patients with CD4+ < 100 cells/μL.

CrAg prevalence estimated in our patient sample was somewhat lower than that reported from other countries in the region [22, 23, 24] Inclusion of ART-experienced patients and incomplete retrieval of all eligible samples may have lowered the estimated prevalence of CrAg in our study, even though similar studies including ART-experienced patients have shown high CrAg prevalence [25, 27]. The relatively low prevalence that we encountered could potentially underestimate the true prevalence of CrAg, and therefore the potential value of a screen and treat program. Nevertheless, our results demonstrate that CrAg prevalence among patients with CD4<100 cells/μL is *at least* high as the threshold of 3% recommended by the WHO to justify routine screening. Our relatively low prevalence may also be attributable to different methodologies and tests used for assessing prevalence; differences in host factors; or geographic variations in the epidemiology of CM or latent infection with Cryptococcus within sub-Saharan Africa region. Our study was not powered to make subnational comparisons in CrAg prevalence. Given the expected ecological variation in CrAg, subnational comparisons in prevalence could have provided useful information to programs about where screening programs would be most effective.

Previous studies have found that CrAg is uncommon among patients with a CD4^+^ >100 cells/μL [28, 29, 30, 31]. Our study found no significant differences in prevalence estimates between patients with CD4^+^ above and below the 100 cells/μL threshold. This result suggests potential benefits of including patients with CD4^+^ 100–200 cells/μL in routine CrAg screening guidelines as suggested by a recent study from Tanzania [32]. However, the results of our statistical comparison should be interpreted with caution because the limited sample size reduced the precision of the prevalence estimates overall and by sub-group. Therefore, routine screening and preemptive treatment should be prioritized among the patients with CD4^+^ <100 cells/μL. This recommendation is substantiated by the significantly higher prevalence of CrAg among patients with a CD4^+^ < 50 cells/μL.

## Conclusions

Reducing mortality from HIV-related CM has long been a focus of HIV care and treatment programs. Recently the focus has shifted from improving CM treatment to preventing symptomatic CM through early detection and preemptive treatment because prognosis is poor after the onset of clinical symptoms. Prior to publication of this paper, the results were used by the MOHSS and its partners in the revision of national ART guidelines (2014), which now include routine screening for CrAg and preemptive treatment for adults with CD4^+^ < 100 cells/μL. The Namibian 2014 ART guidelines include routine screening for ART-naïve and ART-experienced patients. Beginning in February 2014, HIV care and treatment providers across the country began implementing the new screening guidelines. Other national HIV programs should consider similar assessments and synthesis of the results to inform policy and guidelines. Finally, findings from observational studies in the southern African region—which demonstrate the potential for poor patient outcomes even when screening programs are in place. Routine screening programs should be coupled with additional assessments intended to identify more effective, affordable and deliverable regimens for cryptococcal meningitis in order to maximize [33, 34].

## Supporting Information

S1 Table Select demographic and clinical characteristics of sampled patients, sero-survey of *Cryptococcus* antigenemia among HIV-infected adults with advanced immunosuppression in Namibia, 2013–14. ^a.^ data missing, n = 11 ^b.^ data missing, n = 12 ^c.^ data missing, n = 12.
(PDF)

S2 Table Prevalence and correlates of CrAg positivity among HIV-infected adults with advanced immunosuppression in Namibia, 2013–14. a. 95% confidence intervals are binomial exact, two-sided CI interval where no cases of CrAg were detected.
(PDF)

S1 Text Data file. This supporting file is the analytic dataset.
(DTA)
