# Supplementary material for: Estimated Prevalence of Cryptococcus Antigenemia (CrAg) among HIV-Infected Adults with Advanced Immunosuppression in Namibia Justifies Routine Screening and Preemptive Treatment
Source: PLoS One. 2016 Oct 19;11(10):e0161830. doi: 10.1371/journal.pone.0161830 (PMC5070823; doi:10.1371/journal.pone.0161830)
Supplement: S1 Table — a. data missing, n = 11 b. data missing, n = 12 c. data missing, n = 12. (PDF) [file pone.0161830.s001.pdf]

Table 1. Select demographic and clinical characteristics of sampled patients, sero-survey of *Cryptococcus* antigenemia among HIV-infected adults with advanced immunosuppression in Namibia, 2013-14.

Variable	Result
**Patient samples included**, #	825
**Sex**, *#, [% (95% CI)]* [a]	
Female	374, [45.9 (42.5 – 49.4)]
Male	440, [54.1 (50.6 – 57.5)]
**Age***, median (min - max) (IQR) years* [b]	38 (15 - 94) (32 - 46)
**Age group**, *#, [% (95% CI)]*	
15 - 24 years	50 [6.2 (4.7 – 8.0)]
25 - 34 years	224 [27.6 (24.6 – 30.7)]
≥ 35 years	539 [66.3 (63.0 – 69.5)]
**$CD4^+$ result***, median (IQR) cells/µL* [c]	85 (51 - 114)
**$CD4^+$ result strata 1***, #, [% (95% CI)]*	
< 100 cells/µL	511, [62.9 (59.7 -66.4)]
100 - 200 cells/µL	302, [37.1 (33.9 – 40.5)]
**$CD4^+$ result strata 2***, #, [% (95% CI)]*	
< 50 cells/µL	194, [23.5 (20.7 – 26.6)]
50 - 200 cells/µL	630, [76.5 (73.4 – 79.3)]

[a.] *data missing, n=11*

[b.] *data missing, n=12*

[c.] *data missing, n=12*
