# Supplementary material for: Estimated Prevalence of Cryptococcus Antigenemia (CrAg) among HIV-Infected Adults with Advanced Immunosuppression in Namibia Justifies Routine Screening and Preemptive Treatment
Source: PLoS One. 2016 Oct 19;11(10):e0161830. doi: 10.1371/journal.pone.0161830 (PMC5070823; doi:10.1371/journal.pone.0161830)
Supplement: S2 Table — a. 95% confidence intervals are binomial exact, two-sided CI interval where no cases of CrAg were detected. (PDF) [file pone.0161830.s002.pdf]

Table 2. Prevalence and correlates of CrAg positivity among HIV-infected adults with advanced immunosuppression in Namibia, 2013-14.

Variable	CrAg Prevalence (95% CI) [a]	Standard error	Relative standard error	*P*-value
**Overall**	3.3 (2.3 – 4.9)	0.6	18.6	
**Sex**				
Female	2.9 (1.6 – 5.2)	0.9	29.7	0.58
Male	3.6 (2.2 – 5.9)	0.9	24.6	
**Age**				
15 - 34 years	4.0 (2.2 – 7.1)	1.2	29.6	0.43
$\geq$ 35 years	3.0 (1.8 – 4.8)	0.7	24.7	
**CD4^{+} count strata 1** ,				
< 100 cells/µL	3.9 (2.5 – 6.0)	0.9	21.9	0.22
100 - 200 cells/µL	2.3 (1.1 – 4.8)	0.9	37.4	
**CD4^{+} count strata 2**,				
< 50 cells/µL	7.2 (3.6 – 11.3)	1.8	24.4	0.001
50 - 200 cells/µL	2.2 (1.5 – 2.9)	0.3	14.2	

a. 95% confidence intervals are binomial exact, two-sided CI interval where no cases of CrAg were detected.
